# Unresectable intrahepatic cholangiocarcinoma: TARE or TACE, which one to choose?

**DOI:** 10.3389/fgstr.2023.1270264

**Published:** 2023-10-10

**Authors:** Maria Adriana Cocozza, Lorenzo Braccischi, Antonio De Cinque, Antonio Bruno, Alberta Cappelli, Matteo Renzulli, Antonello Basile, Massimo Venturini, Pierleone Lucatelli, Francesco Modestino, Cristina Mosconi

**Affiliations:** ^1^ Department of Radiology, IRCCS Azienda Ospedaliero-Universitaria di Bologna, Bologna, Italy; ^2^ Interventional Neuroradiology Unit, University Hospital of Parma, Parma, Italy; ^3^ Department of Experimental, Diagnostic and Interventional Radiology Unit, “C.A. Pizzardi” Maggiore Hospital, Bologna, Italy; ^4^ Radiology I Unit, Policlinico Universitario G.Rodolico, Catania, Italy; ^5^ Diagnostic and Interventional Radiology Department, Circolo Hospital, ASST Sette Laghi, Insubria University, Varese, Italy; ^6^ Vascular and Interventional Radiology Unit, Department of Diagnostic Service, Sapienza University of Rome, Rome, Italy

**Keywords:** intrahepatic cholangiocarcinoma, TACE, TARE, radioembolization, intrarterial therapies

## Abstract

Intrahepatic cholangiocarcinoma (ICC) is the second most common primary liver malignancy and its incidence is rising in Western countries. Although surgical resection is considered the only curative treatment, up to 70% of patients are diagnosed at an advanced stage, which precludes surgical intervention. Those who are inoperable become candidates for systemic treatment. Currently, the combination of gemcitabine and cisplatin is the first-line chemotherapy, with a median overall survival (OS) of about one year. Recently, there has been a notable increase in evidence regarding chemotherapy for biliary tract cancer; however, the effectiveness of the new chemotherapy drugs still needs to be evaluated. Today, intra-arterial therapies (IAT), especially trans-arterial chemoembolization (TACE) and trans-arterial radioembolization (TARE), are widely used. Both TACE and TARE have demonstrated good efficacy in controlling localized disease and in improving survival. However, current literature does not conclusively show whether TACE is superior to TARE or vice versa. As recent meta-analyses have indicated, both TACE and TARE offer suboptimal objective response rates but yield similar positive outcomes. It’s important to note that these findings are based on single-center studies, which often include a small number of patients and lack a comparative design. Therefore, when comparing such studies, there’s an inevitable selection bias among the treatment groups (TACE or TARE) and significant heterogeneity. This review outlines the current evidence on the use of interventional IAT in managing ICC.

## 1 Introduction

Intrahepatic cholangiocarcinoma (ICC) is a rare and aggressive type of liver cancer that originates from the biliary tract epithelium within the liver. It constitutes approximately 10-20% of all primary liver cancers and carries a poor prognosis, with a median survival of less than a year for advanced cases. Over the past few decades, the incidence of ICC has been on the rise globally, and the reasons for this trend remain unclear (1, 2).

There are several treatment options for ICC, including surgery, chemotherapy, locoregional therapies, and the newer immunotherapies. Locoregional therapies comprise transarterial chemoembolization (TACE) and transarterial radioembolization (TARE), both of which are categorized as intra-arterial therapies (IAT). However, the optimal treatment strategy for ICC is still debated, and a consensus on the most effective method is lacking (3, 4).

Often, patients with ICC are diagnosed when they present with symptoms or when their laboratory tests reveal abnormal values. Nonetheless, asymptomatic cases are sometimes detected incidentally during radiological examinations performed for other medical reasons. Owing to this, up to 70% of ICC cases are diagnosed at advanced stages, limiting treatment options (5, 6). While surgery is the preferred treatment for early-stage ICC, it’s frequently not an option in advanced cases due to tumor location, the presence of multiple lesions, vascular invasion, or metastasis. Furthermore, ICC is generally viewed as a contraindication for liver transplantation. However, post-2014 retrospective studies suggest that carefully selected patients with very early-stage ICC might benefit from liver transplantation (7).

Chemotherapy for ICC can be applied as either a neoadjuvant or adjuvant regimen, or for patients deemed unresectable. The intent behind neoadjuvant chemotherapy is to mitigate the risk of early recurrence or to downstage borderline cases. Yet, given its low efficacy, there’s no established recommendation for neoadjuvant chemotherapy in ICC. On the other hand, capecitabine is recommended as the standard care post-surgery for ICC, based on the BILCAP trial (8, 9).

For advanced-stage ICC patients not eligible for locoregional or surgical treatment, chemotherapy has been widely researched. However, the outcomes are often disheartening, with limited survival benefits and low response rates (6, 9). Gemcitabine combined with cisplatin is the most prevalent first-line chemotherapy regimen, but it yields a median progression-free survival of only 8 months and a median overall survival of less than a year in advanced cases (10).

Immunotherapy, an innovative treatment, seeks to activate the patient’s immune system to recognize and eliminate cancer cells. Numerous studies are underway to determine the safety and effectiveness of immunotherapies, especially in combination with other drugs, for patients with advanced stages. Several strategies aim to enhance T cell activation, reduce immunosuppressive elements, present more tumor-associated antigens, or modify the immunological environment to foster an immune response. However, the efficacy of immunotherapy for ICC remains uncertain, with clinical trials yielding varied outcomes (11, 12).

Another viable option for advanced ICC stages is IAT. TACE and TARE, minimally invasive locoregional procedures, have displayed encouraging results in treating ICC. TACE involves administering a chemotherapeutic agent directly to the tumor through the hepatic artery and subsequently embolizing the tumor’s arterial blood supply. In contrast, TARE delivers radioactive microspheres directly to the tumor via the hepatic artery, emitting radiation to destroy cancer cells while preserving healthy liver tissue (13, 14).

Both TACE and TARE have shown efficacy in treating ICC, either as independent treatments or in combination with other modalities. Currently, however, there’s insufficient evidence to assert the superiority of one over the other. Much of the existing literature stems from individual, single-center studies, often characterized by limited sample sizes and non-comparative designs. Indeed, analyses often conclude that the choice between the two procedures hinges on precise patient selection and the judicious use of available resources. Additionally, no conclusive evidence suggests IATs are superior to conventional treatments (9, 15).

Contemporary guidelines, especially the recent updates from ESMO and EASL-ILCA, highlight the role of IATs in managing patients with non-metastatic disease who are not candidates for surgery. Paired with chemotherapy, TACE and TARE can enhance response and disease control, presenting a viable alternative for patients with unresectable disease and no extrahepatic lesions (16, 17).

Our current review seeks to scrutinize every available study on IAT for unresectable ICC, documenting various indications and responses for TACE and TARE. By assimilating the latest data and employing a meta-analysis approach, we endeavor to discern whether a specific IAT demonstrates superiority over other treatments in terms of quantitative response, clinical side effects, and overall survival.

## 2 Methods

### 2.1 Literature search strategy

A systematic literature review was conducted on PubMed and EMBASE to investigate IAT in ICC until 1 March 2023. Moreover, a check of the Cochrane Central Register of Controlled Trials was performed. Subsequently, we search the following terms: “cholangiocarcinoma”, “bile duct neoplasms”, “embolization, therapeutic”; “chemoembolization, therapeutic”, “Yttrium radioisotopes”, “radioembolization” and “chemoembolization”.

### 2.2 Literature screening

Firstly, an author (M.A.C.) conducted an initial screening to evaluate and remove duplicates from the PubMed and Web of Science. Subsequently, a review of the articles was performed to exclude papers not pertinent or relevant, on the basis of the title or abstract. To reduce potential bias, two other authors (L.B. and A.B.) independently examined the final list of studies, evaluating its adequacy. Only papers with data about overall survival, clinical adverse events (not including biochemical toxicities) and tumour overall response rate (complete response + partial response) after IAT were considered.

**Figure 1:**
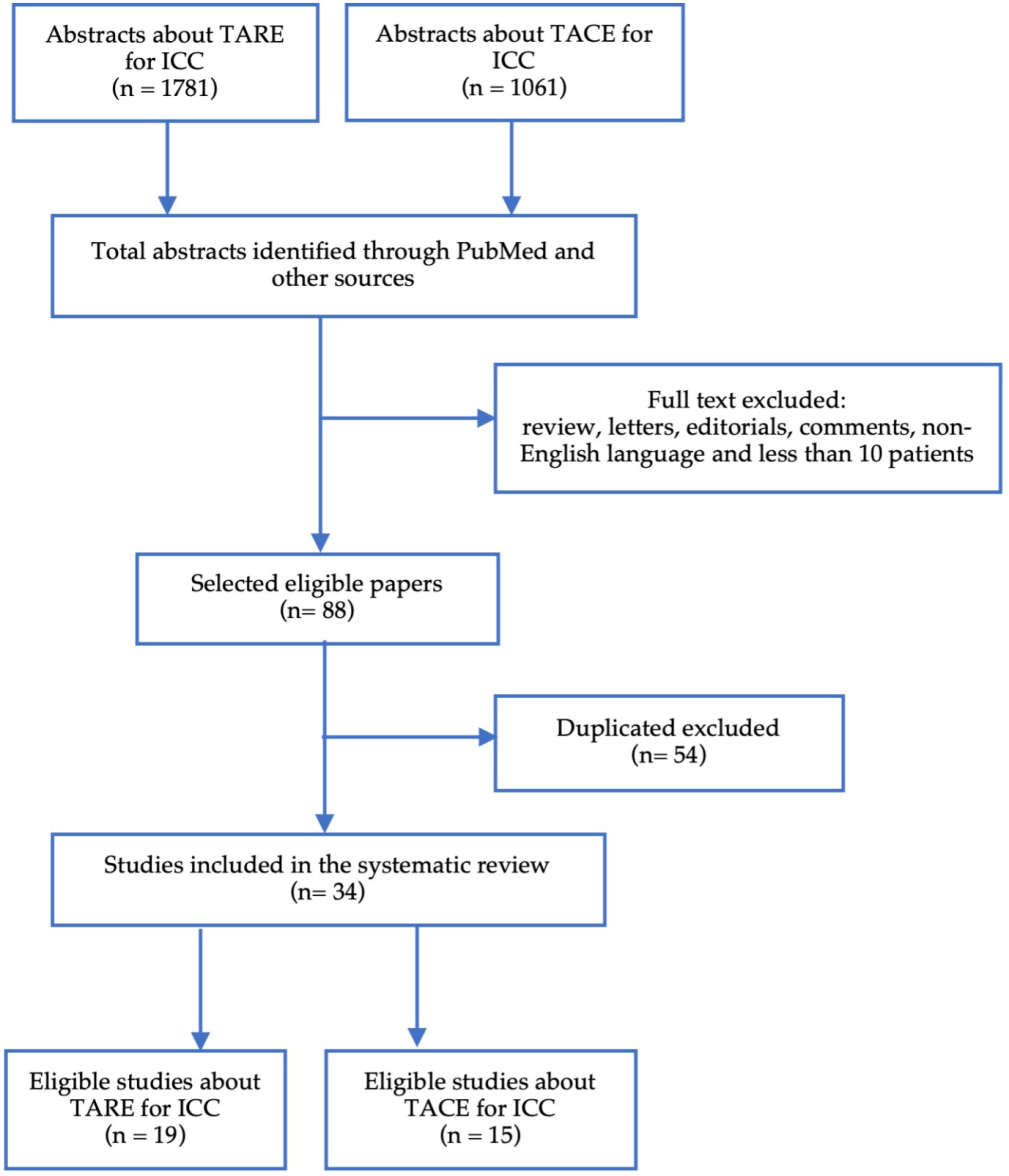
PRISMA flow-chart of selection of the studies included.

The research identified 2842 abstracts, of which 34 entries were eligible (**Figure 1**). The main exclusion criteria were reviews, duplicates, number of patients less than 10 and the absence of outcome data on IAT in unresectable ICC.

## 3 Results

### 3.1 Transarterial chemoembolization

Both conventional TACE (cTACE) and drug-eluting bead TACE (DEB-TACE) demonstrated their efficacy in the ICC treatment; the principal studies about TACE in ICC are reported in **Table 1** (18–32) and shows a median OS of 13.3 months with results in terms of objective response and adverse events of 22.7% and 54.5%, respectively.

**Table 1:** Summary of clinical features and outcomes retrieved from systematic search of TACE in the treatment of intra-hepatic cholangiocarcinoma.

Author (year)	IAT	Pts.	Age (mean. SD)	Male (%)	PS01 (%)	CHT (%)	EHD (%)	Naïve (%)	Burden >25% (%)	Bilobar (%)	Prospective Design	Objective Response (%)	Clinical Adverse Events (%)	Median OS (%)
Martin (2022) (18)	TACE	24	NA	50	100	100	29	100	54.2	58	Yes	50	34	33.7
Sun (2021) (19)	DEB-TACE	40	61.8 (10.7)	62.5	100	10	77.5	47.5	NA	NA	No	67.5	NA	10
Sun (2021) (19)	TACE	49	57.4 (10.4)	57.1	100	12.2	59.2	51.0	NA	NA	No	57.1	NA	6
Ge (2020) (20)	TACE	183	55.9 (10.0)	60.1	NA	0.0	13.7	0.0	NA	NA	No	NA	NA	26.9
Goerg (2019) (21)	DEB-TACE	21	61.3 (13.5)	23.8	100	0.0	0.0	57.1	52.4	52.4	No	61.1	23.8	13.3
Wright (2018) (22)	cTACE	41	62.3 (9.7)	53.7	NA	0.0	56.1	NA	NA	90.2	No	NA	NA	16
Pandey (2018) (23)	cTACE	111	62.0 (12.0)	36.0	NA	NA	39.6	NA	NA	NA	No	NA	NA	17
Aliberti (2017) (24)	DEB-TACE	127	64.5 (7.7)	39.4	NA	NA	0.0	60.6	NA	NA	No	15.0	NA	14.5
Lu (2017) (25)	cTACE	75	56.0 (11.0)	NA	NA	NA	80.0	NA	NA	NA	No	NA	81.3	22.5
Scheuerman (2013) (26)	cTACE	32	64 (10.4)	53.1	NA	NA	0.0	NA	NA	59.4	No	NA	21.9	11
Kuhlmann (2012) (27)	cTACE	10	61.3 (17.8)	80.0	NA	0.0	40.0	70.0	NA	NA	No	12.5	100.0	5.7
Kuhlmann (2012) (27)	DEB-TACE	26	67.0 (7.6)	57.7	NA	0.0	42.3	57.7	NA	NA	No	4.0	100.0	11.7
Vogl (2012) (28)	cTACE	115	60.4 (9.8)	52.2	NA	NA	0.0	NA	NA	77.4	No	8.7	NA	13
Park (2011) (29)	cTACE	72	63.9 (10.1)	65.3	97.2	NA	54.2	NA	NA	51.4	No	22.7	23.6	12.2
Kiefer (2011) (30)	cTACE	62	62.0 (11.2)	40.3	98.4	0.0	30.6	56.5	NA	NA	No	11.1	8.1	15
Shitara (2008) (31)	DEB-TACE	20	69.3 (8.7)	50.0	75.0	NA	85.0	85.0	NA	NA	No	50.0	75.0	14.1
Aliberti (2008) (32)	DEB-TACE	11	68.5 (6.9)	NA	NA	NA	NA	0.0	NA	63.6	No	NA	100.0	13

IAT, intra-arterial therapy; Pts., patients; PS01, Performance Status 0 or 1; CHT, associated chemotherapy; EHD, extra-hepatic disease; NA, not available.

#### 3.1.1 cTACE

**Figure 2:**
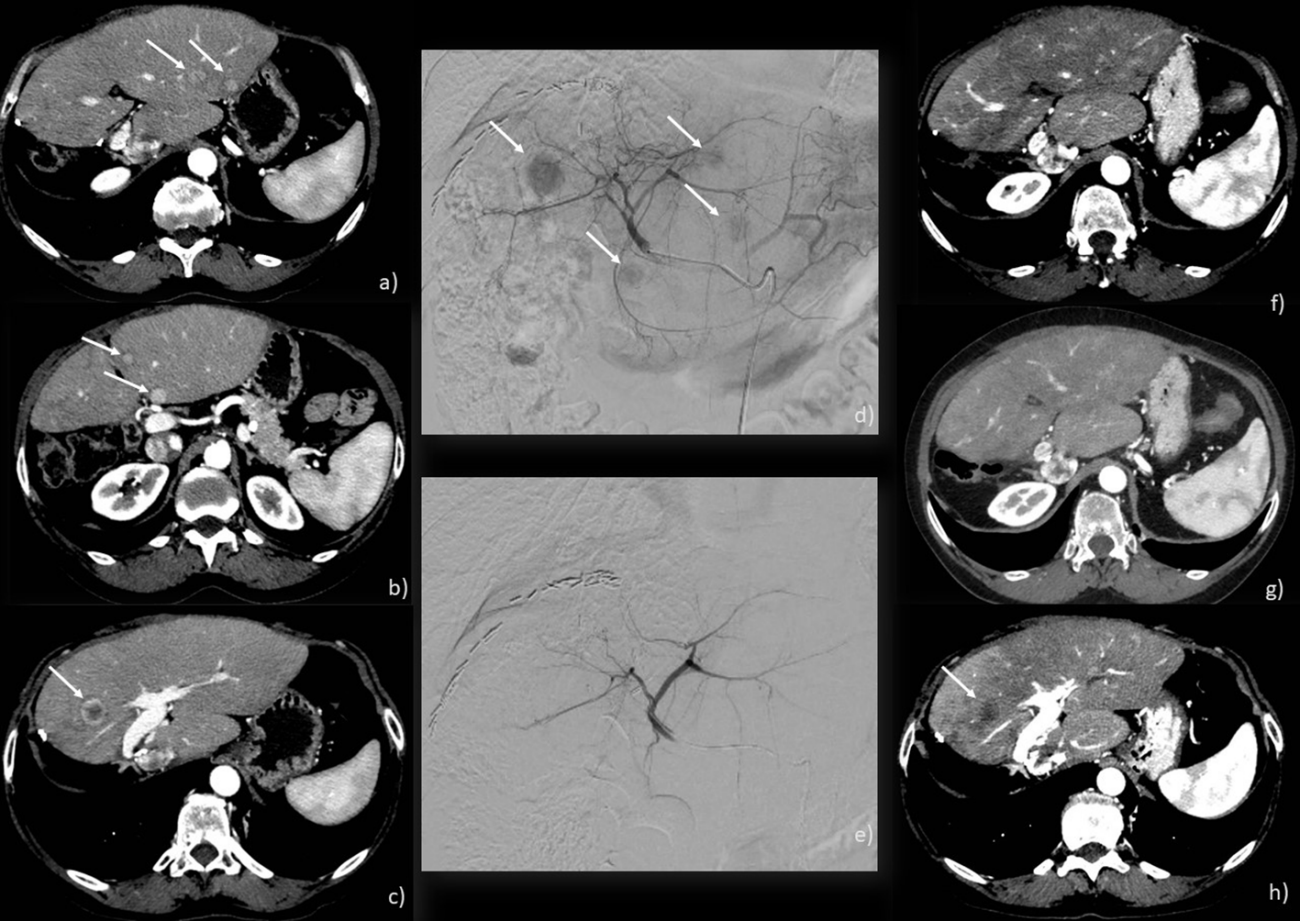
c-TACE performed on a 71-year-old woman: **(A–C)** CT scan showing 4 ICC nodules in both liver lobes (arrows); **(D)** angiographic study showing ICC nodules (arrows); **(E)** angiographic study after injection of chemotherapeutic showing the exclusion of the feeding vessels to the lesions; **(F–H)** CT scan post-treatment showing a good response with necrosis of the main lesion (arrow) and no new emerging lesions.

cTACE is indeed the most common intra-arterial treatment for ICC. It involves injecting an emulsion of chemotherapeutic agents and an oil-based iodinated contrast medium (such as Ethiodol or Lipiodol), followed by the introduction of an embolizing agent into the tumor’s feeding artery (**Figure 2**). In the United States and Europe, a combination of doxorubicin, cisplatin, and mitomycin-C is widely used, although gemcitabine has also been employed (30, 33). The primary inclusion criteria for cTACE in ICC patients are good liver function and an adequate performance status [0–2].

One of the pioneering studies on cTACE in ICC was published in 2011 (29). The authors compared the clinical outcome and survival of cTACE to supportive therapy in 155 patients. In the TACE group, 23% of patients achieved a partial response (PR), and their overall survival (OS) was notably longer (median 12.2 months) than those receiving symptomatic treatment (median 3.3 months, p <0.001). A multicenter study (30) involving 62 patients treated with cTACE reported an overall survival of 15 months from TACE initiation and 20 months from the initial diagnosis, and a time to progression (TTP) of roughly 8 months. According to RECIST criteria, 10% of cases showed PR, while in 66%, the disease remained stable. These findings contrast with those reported by Kuhlmann et al., who found no significant benefit of cTACE in ICC patients, noting 5.7 months of OS and only 1.8 months of progression-free survival (PFS) (27).

Interestingly, Scheuermann et al., in 2013, found no survival difference between patients who underwent non-radical surgical therapy and inoperable patients treated with TACE (17). This observation was later corroborated by Wright et al. (22), suggesting that surgery doesn’t confer a survival advantage over IATs in non-monofocal ICCs. A 2020 study also highlighted that cTACE might offer better survival benefits than percutaneous microwave coagulation therapy (20). Both treatments provided curative outcomes, but TACE had a more pronounced survival advantage (26.9 vs. 12.1 months, p=0.034).

A significant review noted that inoperable ICC patients undergoing cTACE had survival rates ranging from 12 to 25.2 months post-diagnosis and 9.1 to 16.3 months post-treatment (34). Numerous meta-analyses on TACE have shown varied increases in survival. Still, there’s a consensus that TACE treatments generally lead to enhanced survival compared to systemic therapies. The primary goal of TACE is often palliative, and few studies have assessed its role as a downstaging tool, primarily due to experimental design constraints.

However, the existing literature varies significantly in terms of lesion number and size, the chemotherapeutic agent chosen, and the number of treatment sessions. A meta-analysis by Ray et al. (35) focusing on chemotherapy-based transarterial therapies determined that the cumulative median OS from the diagnosis date and from the first TACE session were 15.7 months and 13.4 months, respectively. Despite the study’s limitations and potential biases, in the absence of randomized controlled trials, these findings serve as a reasonable benchmark for TACE’s efficacy and safety (36).

Generally, cTACE is well-tolerated by patients, and severe adverse events are rare. The most common minor adverse effect is the post-embolization syndrome, characterized by symptoms like fever, a temporary spike in liver enzymes, nausea, and pain (37). Currently, there’s no significant difference in complication rates between cTACE and DEB-TACE.

#### 3.1.2 DEB-TACE

DEB-TACE technique is built on the principle of combining embolization with drug release. This approach provides the opportunity for continuous release of the chemotherapeutic agent(s) into the tumor area, allowing for controlled drug release and dosage (32). The literature on DEB-TACE is more limited compared to cTACE. The initial study by Aliberti et al. reported a median survival of 13 months with a favorable response; these findings were confirmed by a more recent paper from the same group (OS=14.5 months) (32). Reported survival durations post-treatment with DEB-TACE in patients with inoperable ICC range from 8.6 to 30 months (34).

Recently, a comparative study on the efficacy and safety of DEB-TACE versus cTACE for treating unresectable intrahepatic cholangiocarcinoma patients was conducted by Sun et al.’s group (19). DEB-TACE demonstrated a significant improvement in OS compared to cTACE (median OS of 10 months vs 6 months, P=0.006) and was well-tolerated. These findings are consistent with the latest literature reviews (38, 39). However, as with cTACE, there are concerns regarding the heterogeneity of the reported data, especially in terms of lesion number and size, the chosen chemotherapeutic agent, and treatment sessions.

The combination of IAT with chemotherapy is a topic of great interest. A recent randomized phase II study by Martin et al. assessed the efficacy of irinotecan drug-eluting beads IAT in combination with systemic therapy (Gem/Cis) against Gem/Cis alone in patients with unresectable ICC (18). The results were significantly better in the combined therapy group compared to the solo therapy group (overall response rate at 2 (p < 0.04), 4 (p < 0.03), and 6 months (p < 0.05); downsizing to resection/ablation was 25% versus 8%, p < 0.05; median progression-free survival was 31.9 (95% CI 8.5-75.3) months versus 10.1 (95% CI 5.3-13.5) months, p = 0.028; OS was 33.7 (95% CI 13.5-54.5) months versus 12.6 (95% CI 8.7-33.4) months, p = 0.048). The combined approach proved to be safe and resulted in significant downsizing to resection, with improved progression-free survival and overall survival.

## 4 Transarterial radioembolization

**Figure 3:**
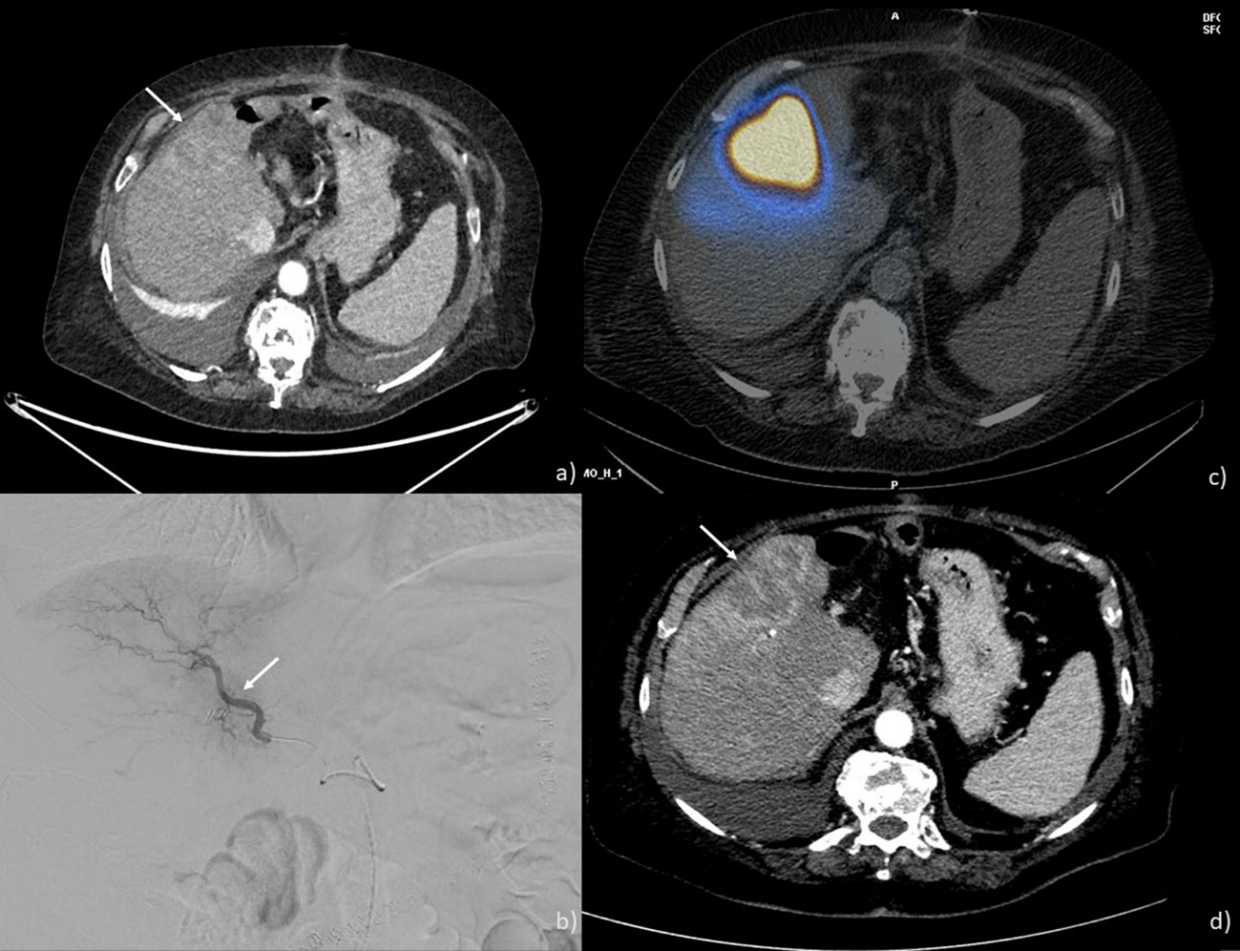
TARE performed on a 69-year-old man: **(A)** CT scan showing an ICC nodule in the 5th and 8th hepatic segments (arrow); **(B)** angiographic study showing ICC nodule the placement of the microcatheter at the level of the branch for the anterior segments (arrow); **(C)** SPECT-CT after Tc-99m macro aggregated albumin injection showing a good coverage of the lesion **(D)** CT scan post-treatment showing a good response with necrosis of the lesion (arrow).

Radioembolization involves the intra-arterial injection of radioactive microspheres to selectively release high levels of radiation specifically to the ICC, while sparing healthy liver tissue from radiation (**Figure 3**). The best candidates for radioembolization are those with unresectable liver-only or liver-dominant tumors (40). Inclusion criteria encompass having an unresectable tumor, an ECOG PS of 0, 1, or 2, and good liver function. Exclusion criteria include flow to the gastrointestinal tract that’s ineligible for embolization with a coil and a single administration radiation dose to the lungs exceeding 30 Gy.

Key studies on TARE in ICC are summarized in **Table 2** (41–61), indicating a median OS of 14.2 months, with objective response rates and adverse events at 25.0% and 38.4%, respectively.

**Table 2:** Summary of clinical features and outcomes retrieved from systematic search of Y90 in the treatment of intra-hepatic cholangiocarcinoma.

Author (year)	IAT	Pts	Age (mean. SD)	Male (%)	PS01 (%)	CHT (%)	EHD (%)	Naïve (%)	Burden >25% (%)	Bilobar (%)	Prospective Design	Objective Response (%)	Clinical Adverse Events (%)	Median OS (months)
Mosconi (2023) (41)	Y90	49	62 (12.0)	47.0	100	43.0	33.0	NA	NA	57.0	No	64.6	28.6	16.0
Gupta (2022) (42)	Y90	136	NA	51.0	96.0	41.9	36.0	86.0	31.0	58.0	No	24.4	1.5	14.2
Robinson (2022) (43)	Y90	95	66 (11.0)	53.0	91.0	74.0	27.0	NA	NA	54.0	No	34.0	4.1	14.0
Paprottka (2021) (44)	Y90	73	NA	55.0	NA	71.0	51.0	NA	40.0	NA	No	25.0	12.0	11.8
Edeline (2020 (45)	Y90	41	64.0 (10.7)	63.4	100	100.0	0.0	100.0	NA	34.1	Yes	42.5	70.7	22.0
Buettner (2020) (46)	Y90	115	NA	47.8	93.0	0.0	23.5	20.9	NA	71.3	No	7.1	25.2	29.0
Bargellini (2020) (47)	Y90	81	62.4 (11.8)	60.5	100	0.0	24.7	43.2	48.1	49.4	No	41.8	0.0	14.5
Köhler (2019) (48)	Y90	46	60.5 (14.0)	41.3	NA	0.0	30.4	34.8	63.0	50.0	No	34.8	0.0	9.7
White (2019) (49)	Y90	61	63.8 (4.0)	52.5	90.2	11.5	36.1	8.2	NA	63.9	Yes	13.0	49.2	8.7
Levillain (2019) (50)	Y90	58	65.0 (12.0)	39.7	100	0.0	NA	0.0	NA	56.9	No	NA	NA	10.3
Reimer (2018) (51)	Y90	21	69.5 (8.5)	57.1	14.3	0.0	14.3	100.0	61.9	19.0	No	4.8	4.8	
Gangi (2018) (52)	Y90	85	73.4 (9.3)	48.2	67.1	0.0	42.4	22.4	NA	36.5	No	6.2	48.2	12.0
Shaker (2018) (53)	Y90	17	69.3 (11.0)	41.2	NA	0.0	41.2	52.9	NA	NA	No	NA	11.8	33.6
Bourien (2018) (54)	Y90	64	NA	57.8	89.1	51.6	15.6	56.3	NA	56.3	No	14.5	90.6	16.4
Soydal (2016) (55)	Y90	16	55.4 (17.7)	50.0	100	56.3	31.3	25.0	75.0	50.0	No	31.3	NA	9.6
Filippi (2015) (56)	Y90	17	59.4 (10.5)	35.3	100	88.2	23.5	11.8	23.5	17.6	Yes	NA	52.9	14.7
Camacho (2014) (57)	Y90	21	61.2 (9.8)	61.9	81.0	100.0	NA	0.0	NA	NA	Yes	0.0	NA	16.3
Mouli (2013) (58)	Y90	46	66.5 (10.7)	54.3	97.8	NA	34.8	39.1	21.7	34.8	Yes	NA	54.3	
Rafi (2013) (59)	Y90	19	63.3 (15.1)	36.8	78.9	NA	57.9	0.0	NA	42.1	Yes	10.5	89.5	11.3
Hoffmann (2012) (60)	Y90	33	65.2 (10.2)	54.5	72.7	NA	24.2	0.0	45.5	63.6	No	36.4	84.8	22.0
Saxena (2010) (61)	Y90	25	57.0 (12.0)	52.0	88.0	NA	48.0	24.0	60.0	80.0	Yes	26.1	64.0	9.3

IAT, intra-arterial therapy; Pts., patients; PS01, Performance Status 0 or 1; CHT, associated chemotherapy; EHD, extra-hepatic disease; NA, not available.

Ibrahim et al. published a study on TARE in 24 inoperable ICC patients. According to the WHO Criteria (with follow-up data available for 22 patients), a PR was observed in 27% of cases, stable disease (SD) in 68% of patients, and disease progression (PD) in 5% (62). The reported median OS from the time of the first treatment was 14.9 months. Notably, patients with an ECOG PS of 0 experienced a significantly better OS than those with a PS of 1 or 2. Moreover, survival rates varied significantly based on the presence of portal vein thrombosis and the type of tumor. Several other studies, including those by Mouli et al. and Hoffmann et al., have reported favorable outcomes with TARE treatment, emphasizing factors like ECOG status, tumor location, and tumor response as significant determinants of survival (58, 60).

Saxena et al.’s study, encompassing 25 patients, found a 74% disease control rate according to the RECIST Criteria, with a median OS of 9.3 months (61). Meanwhile, a study by Rafi et al. reported a median survival of roughly 345 days and indicated that performance status and the presence of extra-hepatic disease were not significantly correlated to survival (45).

A multicenter, prospective observational registry encompassing 27 centers and 95 patients showcased promising results, emphasizing the overall positive role of TARE in improving survival (43). Additionally, several studies and reviews have attempted to determine predictive factors for OS and TARE response to better delineate eligible patients (42, 44, 57, 63–65).

In particular, Edeline et al.’s phase 2 clinical trial highlighted the efficacy of combining first-line chemotherapy (cisplatin and gemcitabine) with TARE, showing substantial OS improvements (66). Yet, a retrospective multicenter study with 81 patients yielded differing results, suggesting that the sequence and combination of therapies can significantly influence outcomes (47).

Like TACE, TARE is a well-tolerated treatment with few major adverse events. Common minor side effects include abdominal pain, nausea, fever, and an increase in liver enzymes (64).

## 5 Discussion: TARE or TACE, which one to choose?

The prognosis of ICC is poor, as only 15 to 30% of cases are deemed resectable; without treatment, the median OS stands at 3 months. The National Comprehensive Cancer Network (NCCN) guidelines advocate for chemotherapy in unresectable ICC, especially the combination of cisplatin and gemcitabine. However, this regimen is linked to a median survival of roughly 11.6 months (10). Given the limited survival benefits associated with chemotherapy, various local regional therapies, particularly IAT, have been explored. Nevertheless, due to the rarity of ICC, studies on IAT are predominantly retrospective, involve small patient cohorts, and lack the robustness needed to deliver definitive recommendations. Though locoregional techniques in ICC have shown promise, they are still categorized as Category 2B by the NCCN guidelines. In this review, both infusion into the hepatic artery and bland embolization are omitted due to the limited number and quality of available papers. Instead, this paper exclusively evaluates TACE and TARE, aligning with a prior decision-making paper which reviewed these treatments without analytically approaching IAT in ICC (67).

There are no randomized studies concerning TACE and TARE for treating inoperable ICC in current literature, and only two meta-analyses compare their effectiveness. Boehm et al.’s meta-analysis from 2014 (68) reported comparable median OS rates across TARE, conventional chemoembolization, and DEB-chemoembolization. Yet, the authors emphasized the potential for selection bias, mainly because of heterogeneous inclusion criteria across different groups. Another meta-analysis by Edeline’s group (45) discussed how IAT, especially in conjunction with systemic chemotherapy, might be promising in the face of poor outcomes from second-line systemic chemotherapy.

A burgeoning area of interest, meriting further research, is the potential use of IAT as a neoadjuvant therapy for patients with resectable ICC to enhance surgical outcomes. Notably, TARE has demonstrated potential benefits in this context, although no downstaging treatments for ICC are currently recommended (69). Another pivotal aspect to consider in the ICC treatment landscape is the recent advancements in molecular targeted therapies. Encouraging outcomes have emerged from phase II-III clinical trials targeting specific genetic alterations (70–72). In light of this, future studies could aim to pinpoint genetic profiles most responsive to locoregional therapies. However, consensus is lacking in the literature regarding the optimal drug choice for TACE in ICC, in contrast to HCC.

Our analysis echoes the prevailing literature, finding analogous OS rates between TACE and TARE. While the radiosensitivity of ICC is a well-accepted concept in the medical field, TARE hasn’t definitively demonstrated superiority over TACE. The primary hindrance remains the heterogeneous populations studied, emphasizing the importance of identifying eligible patients (73). Presently, the prevailing clinical practice often favors TACE for patients with better PS or those who are treatment-naïve, while TARE is usually selected for recurrent tumors or patients with prior treatments. Such distinctions inevitably skew survival analyses.

Consequently, a thorough comparison between TACE and TARE, taking into account relevant survival parameters, is essential. We eagerly anticipate the results of an ongoing randomized clinical trial comparing these two therapies (74).

Regarding side effects, Boehm et al.’s meta-analysis suggested TACE leads to more severe adverse events. However, accurately comparing complications was challenged by the dearth of data (68). A more recent meta-analysis inferred that chemoembolization results in more pronounced side effects, especially post-embolization syndrome, than TARE (70). Our findings align with this narrative, indicating fewer adverse events for TARE, thus making it preferable prior to biliary interventions and in tandem with systemic chemotherapy. The synergy between chemotherapeutic agents and radiation renders the TARE combination particularly attractive (66).

## 6 Conclusions

To date, as underscored by these findings, there exists a wide variety of indications for IAT, complicating the comparison of long-term outcomes across published studies. The challenges in interpreting these results further complicate the comparison of treatment outcomes between TACE and TARE, making it difficult to pinpoint the most optimal treatment. Excluding adverse events, chemoembolization and radioembolization seem to yield similar OS and response rates.

Given the current literature, until a randomized controlled trial is executed, it’s impossible to definitively determine which therapy outperforms the other and under which conditions. The choice between TACE and TARE should primarily hinge on a center’s resources and the expertise of its practitioners. In institutions where both TACE and TARE are accessible, preference for TARE might lean on the recognized radiosensitivity of ICC. Similarly, as observed in HCC, factors like the size, number, distribution, and vascularization of the nodules might influence practitioners’ choices between the two techniques.

Emerging data are beginning to highlight the role of IAT beyond just palliative care. By downstaging to surgery, the application of locoregional therapies might become more extensive, thereby elevating the significance of IAT in managing ICC. Further investigative studies are crucial to enhance the treatment strategies for ICC in the coming years.

## Data availability statement

The raw data supporting the conclusions of this article will be made available by the authors, without undue reservation.

## Author contributions

MC: Data curation, Formal Analysis, Investigation, Software, Writing – original draft. LB: Data curation, Investigation, Software, Writing – original draft. AD: Data curation, Investigation, Writing – original draft. AB: Supervision, Writing – review & editing. AC: Supervision, Validation, Writing – review & editing. MR: Conceptualization, Methodology, Supervision, Validation, Writing – review & editing. AB: Supervision, Validation, Writing – review & editing. MV: Supervision, Validation, Writing – review & editing. PL: Supervision, Validation, Visualization, Writing – review & editing. FM: Conceptualization, Data curation, Supervision, Validation, Writing – review & editing. CM: Conceptualization, Methodology, Supervision, Validation, Writing – review & editing, Writing – original draft.
